# Differential Yet Integral Contributions of Nrf1 and Nrf2 in the Human HepG2 Cells on Antioxidant Cytoprotective Response against *Tert*-Butylhydroquinone as a Pro-Oxidative Stressor

**DOI:** 10.3390/antiox10101610

**Published:** 2021-10-13

**Authors:** Reziyamu Wufuer, Zhuo Fan, Keli Liu, Yiguo Zhang

**Affiliations:** Laboratory of Cell Biochemistry and Topogenetic Regulation, College of Bioengineering and Faculty of Medical Sciences, Chongqing University, No. 174 Shazheng Street, Shapingba District, Chongqing 400044, China; 20191901703@cqu.edu.cn (R.W.); 18623059592@163.com (Z.F.); 201919021016@cqu.edu.cn (K.L.)

**Keywords:** Nrf1, Nrf2, redox gene regulation, antioxidant, oxidative stress, reactive oxygen species (ROS), *tert*-butylhydroquinone (*t*BHQ), Cap’n’Collar (CNC), basic region-leucine zipper (bZIP)

## Abstract

In the past 25 years, Nrf2 (nuclear factor erythroid 2-related factor 2, also called NFE2L2) had been preferentially parsed as a master hub of regulating antioxidant, detoxification, and cytoprotective genes; albeit as a matter of fact that Nrf1 (nuclear factor erythroid 2-related factor 1, also called NFE2L1)—rather than Nrf2—is indispensable for cell homeostasis and organ integrity during normal growth and development. Herein, distinct genotypic cell lines (i.e., *Nrf1α^−/−^*, *Nrf2^−/−ΔTA^*, and *caNrf2^ΔN^*) are employed to determine differential yet integral roles of Nrf1 and Nrf2 in mediating antioxidant responsive genes to *tert*-butylhydroquinone (*t*BHQ) serving as a pro-oxidative stressor. In *Nrf1α^−/−^* cells, Nrf2 was highly accumulated but also could not fully compensate specific loss of Nrf1α’s function in its basal cytoprotective response against endogenous oxidative stress, though it exerted partially inducible antioxidant response, as the hormetic effect of *t*BHQ, against apoptotic damages. By contrast, *Nrf2^−/−ΔTA^* cells gave rise to a substantial reduction of Nrf1 in both basal and *t*BHQ-stimulated expression levels and hence resulted in obvious oxidative stress, but it can still be allowed to mediate a potent antioxidant response, as accompanied by a significantly decreased ratio of GSSG (oxidized glutathione) to GSH (reduced glutathione). Conversely, a remarkable increase of *Nrf1* expression resulted from the constitutive active *caNrf2^ΔN^* cells, which were not manifested with oxidative stress, whether or not it was intervened with *t*BHQ. Such inter-regulatory effects of Nrf1 and Nrf2 on the antioxidant and detoxification genes (encoding HO-1, NQO1, GCLC, GCLM, GSR, GPX1, TALDO, MT1E, and MT2), as well on the ROS (reactive oxygen species)-scavenging activities of SOD (superoxide dismutase) and CAT (catalase), were further investigated. The collective results unraveled that Nrf1 and Nrf2 make distinctive yet cooperative contributions to finely tuning basal constitutive and/or *t*BHQ-inducible expression levels of antioxidant cytoprotective genes in the inter-regulatory networks. Overall, Nrf1 acts as a brake control for Nrf2’s functionality to be confined within a certain extent, whilst its transcription is regulated by Nrf2.

## 1. Introduction

With the development of science and technology, much more effective compounds with antioxidant properties have been discovered or synthesized insofar as to prevent lipid and protein oxidation [1,2]. Amongst them, *tert*-butylhydroquinone (*t*BHQ) is a well-known small molecule phenolic antioxidant, which is a main metabolite of 3-*tert*-butyl-hydroxyanisole (BHA) in vivo in humans, dogs, and rats, since it is widely used as a preservative in oils and processed foods [3,4]. However, *t*BHQ (and its precursor BHA) is de facto identified as a double-faced compound with both effects to be exerted as an antioxidant and also a pro-oxidant in biological systems [5,6]. Of great note, such a double-bladed sword impact of *t*BHQ is further unraveled by chemoprotective and carcinogenic effects of this compound and its reactive metabolites [6], in addition to its cytotoxicity [3,4]. This is due to the fact that oxidative metabolism of *t*BHQ—by metal-mediated redox cycling and microsomal monooxygenase system (e.g. phase I drug-metabolic enzyme cytochrome P450 1a1 (Cyp1a1)—yields several reactive oxygen species (ROS) and electrophilic intermediates, followed by the formation of reactive glutathione conjugates (e.g., GS- in phase II drug-metabolic reactions by glutathione-*S* transferases (GSTs)). Furthermore, *t*BHQ is also identified to function as a novel ligand of aryl hydrocarbon receptor (AhR) [7], such that it can directly induce the expression of Cyp1a1, an enzyme known to play an important role in the chemical activation of xenobiotics to carcinogenic derivatives. Thereby, it is inferred that the AhR-dependent induction of Cyp1a1 by *t*BHQ represents a positive feedback network so as to promote carcinogenicity, particularly upon its long-term exposure, in the gastrointestinal and liver tissues [6,8].

On another facet, the cytoprotective effect of *t*BHQ on biological systems is revealed by bona fide induction of the endogenous antioxidant and detoxification genes (e.g., those encoding phase II drug-metabolic enzymes) in the response to this food additive [8,9]. The endogenous antioxidant defense is provided predominantly by reduced glutathione (GSH) and other thiol-sensitive signaling molecules (e.g., thioredoxin), which contribute to metabolism of potentially harmful pro-oxidant agents (e.g., xenobiotics) and restore the intracellular redox balance to a steady-state, so that cell homeostasis is rebalanced [5,10]. To this end, the expression of such innate antioxidant biosynthetic and detoxifying enzymes is governed primarily by the Cap’n’Collar (CNC) basic region-leucine zipper (bZIP) family of transcription factors [11,12,13]. Amongst this family, Nrf1 and Nrf2 (both encoded by *Nfe2l1* and *Nfe2l2*, respectively) are two principal regulators for maintaining robust redox homeostasis in mammalian life process [13,14,15,16]. To date, most studies of *t*BHQ-induced antioxidant cytoprotective responses have been focused disproportionately on the redox-sensitive Nrf2 [8,11,17,18,19], rather than the putative redox threshold-setting Nrf1 [20,21,22], since the former Nrf2 was firstly identified as a master regulator of the phase II detoxifying enzyme genes (e.g., *NQO1*, *HO*-*1*, *GSTs*) through their antioxidant response elements (AREs) to the pro-oxidant BHA or its metabolite *t*BHQ [5,23]. The underlying mechanisms for *t*BHQ-stimulated activity of Nrf2 are well documented [11,24,25], but it is less understood whether and/or how the transactivation activity of Nrf1 is induced by the exposure to this chemical.

Although Nrf2 is accepted as a master regulator of ARE-driven cytoprotective gene expression [11,25], it is not essential for normal development and healthy growth, because its global knockout (*Nrf2*^−/−^) mice are manifested with neither any obvious defects nor spontaneous pathological phenotypes (e.g., cancer) [23,26]. In effect, *Nrf2*^−/−^ mice are more susceptible than wild-type mice to chemical carcinogens [27], in addition to oxidative stress [28]. Thereafter, induction of Nrf2 (by *t*BHQ) has thus been recognized as a potential chemopreventive and therapeutic target against cancer [25,29]. To the contrary, the long-term induction of hyperactive Nrf2 is also reconsidered as a potent oncogenic driver with several hallmarks of cancer; this is based on its bona fide tumor-promoting effects and also resistance to chemotherapy [30,31]. Such dual opposing roles of Nrf2 in cancer prevention and progression should be severely taken into account for its bidirectional potentials to be implicated in cancer treatment.

By sharp contrast, Nrf1 is endowed with its innate unique features that are distinctive from Nrf2 [12,32,33], as evidenced by its gene-targeting knockout (*Nrf1*^−/−^) in the mouse to establish distinct animal models with significant pathological phenotypes [16,20,22,34,35,36]. Global knockout of *Nrf1*^−/−^leads to murine embryonic lethality at E6.5 to E14.5, resulting from severe oxidative stress [20,34,35]. This fact implies that loss of Nrf1’s function cannot be compensated by Nrf2, though Nrf2 can also contribute to combinational regulation of antioxidant cytoprotective genes as confirmed by a double knockout *Nrf1*^−/−^::*Nrf2*^−/−^ model [14]. Furtherly, distinct tissue-specific *Nrf1*^−/−^ mice are manifested with typical pathologies, resembling human non-alcoholic steatohepatitis (NASH) and hepatoma [16,22], type-2 diabetes [37], and neurodegenerative diseases [38,39]. Collectively, these demonstrate that mouse Nrf1 (and its isoforms) fulfills an indispensable function in regulating critical genes for maintaining robust redox homeostasis and organ integrity, so that the normal physiological development and growth are perpetuated in life process. However, it is regrettable that these achievements are made mostly from mouse models. Such being the case, the underlying mechanism(s) by which human Nrf1 (or its derived isoforms) also contributes to similar pathophysiological cytoprotective responses remains elusive.

For this reason, we have established three specific-knockout cell lines by gene-editing of human Nrf1 or Nrf2 on the base of HepG2 cells (named *Nrf1α^−/−^*, *Nrf2^−/−ΔTA^*, and *caNrf2^ΔN^*, respectively) [31,40]. Here, these three distinct genotypic cell lines together with wild-type cells were stimulated by *t*BHQ and subjected to a series of experimental interrogation of both basal and inducible expression levels of certain antioxidant, detoxification, and cytoprotective genes. The resulting evidence has been presented by us, revealing that human Nrf1 and Nrf2 can make differential, yet integral, contributions to synergistic regulation of antioxidant and detoxification genes induced by *t*BHQ as a pro-oxidative stressor. Of great note, it is plausible that the presence of Nrf1 determines the basal redox steady-state and normal antioxidant cytoprotective responses against endogenous oxidative damages and apoptosis, albeit Nrf2 is involved in this homeostatic function [12,14,21,31]. This study also provides a better understanding of the inter-regulatory roles of Nrf1 and Nrf2 within the redox control system, except that both factors can exert their specific yet combinational functions in the process. Thereby, such cautions should also be severely taken into account for us to develop new drugs targeting Nrf1 or Nrf2 alone or both in the biomedical translational study.

## 2. Materials and Methods

### 2.1. Cell Lines and Regents

The human hepatomacellular carcinoma (HepG2) cells (wide type, *WT*; i.e., *Nrf1/2*^+/+^) were purchased from American Type Culture Collection (ATCC, Manassas, VA, USA). Three HepG2-derived cell lines with distinct knockout types of *Nrf1α*^−/−^ (with a specific deletion mutant of full-length Nrf1/TCF11 and its derived isoforms), *Nrf2^−/−ΔTA^* (lacking its longer transactivation domain-containing fragment), or ca*Nrf2^ΔN^* (i.e., a constitutive active mutant of Nrf2 that lacks its N-terminal Keap1-binding Neh2 domain) had been established in our laboratory, as described in detail by Qiu et al. [31]. It is also worth mentioning that the authenticity of HepG2 cell line had been confirmed by its authentication analysis and STR (short tandem repeat) typing map (which was carried out by Shanghai Biowing Applied Biotechnology Co., Ltd., Shanghai, China). All these cell lines were, separately, cultured in DMEM (Dulbecco’s Modified Eagle’s Medium) supplemented with 10% (*v*/*v*) FBS (fetal bovine serum) and 100 units/L double-antibiotic (penicillin and streptomycin, Solarbio, Beijing, China) and incubated at 37 °C in a humidified 5% CO_2_ atmosphere.

Subsequently, those experimental cells were treated with *t*BHQ (CAS no.1948-33-0, from Sigma, St. Louis, MO, USA), which is a newly synthesized phenolic antioxidant with the chemical formula of C_10_H_14_O_2_ at molecule weight of 166.22. This compound has completely dissolved in DMSO (dimethyl sulfoxide) to a stocked concentration of 50 mM, and stored at −20 °C before it is experimented. Of note, specific antibody against Nrf1 was made in our laboratory [41]. Besides, other five distinct antibodies against Nrf2 (ab62352), GCLC (ab207777), GCLM (ab126704), HO-1 (ab52947), or GPX1 (ab108427) were obtained from Abcam (Cambridge, UK). Additional three antibodies against NQO1 (D26104), GSR (D220726), or TALDO1 (D623398) were from Sangon Biotech (Shanghai, China), whilst β-actin antibody (TA-09) was from ZSGB-BIO (Beijing, China).

### 2.2. Cell Viability with the MTT Assay

All the indicated experimental cells were digested by trypsin and diluted into a suspension of 5 × 10^4^ cell/mL, before being seeded into 96-well plates (5 × 10^3^ cells/well). After the cells were completely adherent to the plates, they were treated with *t*BHQ at different concentrations (i.e., 0–100 μM) for 24 h, or with 50 μM of *t*BHQ for distinct time periods (i.e., 0–24 h). The cell viability was evaluated by assaying 3-(4,5-dimethylthiazol-2-yl)-2,5-diphenyltetrazolium bromide (MTT, ST1537, Beyotime, Shanghai, China) to form an insoluble product formazan in all living cells. The resulting data were calculated by dividing the experimental absorbance by relevant control values. The results were shown as a percentage of mean *±* SD (*n* = 5 × 3), which are representative of at least three independent experiments, each of which was performed in quintuplicates.

### 2.3. Quantitative RT-PCR Analysis of mRNA Expression

All experimental cell lines growing in logarithmic phases were digested with trypsin and diluted by a complete medium into the suspension of 3.5 × 10^5^ cell/mL. Then equal amounts of cells were inoculated in 6-well plates (3.5 × 10^5^ cells/well) and cultured until being completely adherent. Thereafter, they were treated for different time periods (i.e., 0, 4, 8, 12, 16, 20, or 24 h) with 50 μM of *t*BHQ. Subsequently, total RNA was isolated using a RNA extraction kit (TIANGEN, Beijing, China), 500 ng of which was then subjected to the reaction with reverse transcriptase (Promega, Madison, WI, USA) to synthesize the single strand cDNAs, that served as PCR templates. Lastly, both basal and *t*BHQ-induced mRNA expression levels of those indicated genes were detected by quantitative real-time PCR (RT-qPCR) with each pair of their primers (Table 1).

The RT-qPCR reaction was carried out with GoTaq^®^qPCR Master Mix (Promega, Madison, WI, USA) on a CFX96 instrument (Bio-Rad, Hercules, CA, USA). The specific reaction procedure was followed by all relevant experimental groups, which were first inactivated at 95 °C or 3 min, and then amplified by 40 reaction cycles of 15 s at 95 °C and 30 s at 60 °C. The resulting data were analyzed by the Bio-Rad CFX 96 Manager 3.0 software (Hercules, CA, USA), whilst β-actin expression level served an internal reference control. All the experimental values were further calculated by normalization to the basal values obtained from WT cells that had been treated with *t*BHQ for 0 h (this value of 1 is set). The results of all the examined genes were shown as fold changes (Mean ± SD, *n* = 3 × 3), which are representative of at least three independent experiments being each performed in triplicates.

### 2.4. Western Blotting Analysis of Protein Expression

All those indicated experimental cells were allowed for preparation of a suspension of 3.5 × 10^5^ cell/mL, and seeded into 6-well plates at a final density of 3.5 × 10^5^ cells/well. After the cells were completely adherent to the plates, they were treated with 50 μM *t*BHQ for distinct lengths of time (i.e., 0, 1, 2, 4, 8, 12, 16, 20, and 24 h). After they were collected in a lysis buffer, the proteins were extracted for total lysates, which were further diluted with a 3× loading buffer and denatured by boiling at 100 °C for 10 min. The resulting total proteins from each of experimental groups were subjected to separation by SDS (sodium dodecylsulfate)-PAGE (polyacrylamide gel electrophoresis) gels containing 8% polyacrylamide (to resolve Nrf1 and Nrf2) or 10% polyacrylamide (to resolve GSR, GCLC, GCLM, GPX1, HO-1, NQO1, and TALDO), which were allowed for running at 50 v for 30 min and then changed into 100 v to continue running for 2 h, before being transferred on the PVDF (polyvinylidene fluoride) membrane (Millipore Co., Tullagreen, Ireland) at 200 A for 2 h. After the protein-botted membranes were blocked by 5% skimmed milk for 1 h, they were incubated with each of the indicated primary antibodies at 4 °C overnight, and then re-incubated with the secondary antibody at room temperature for 2 h. The protein blots were developed by the enhanced chemiluminescence as described previously [31]. The intensity of relevant immunoblots was calculated by using the Quantity One software (Bio-Rad Laboratories, Hercules, CA, USA) and also normalized to the value of β-actin as a loading control. The resulting data were also shown as fold changes (Mean ± SD, *n* = 3) relative to the respective controls.

### 2.5. Detection of Cellular ROS and Apoptosis by Flow Cytometry

After a suspension of each of experimental cell lines were prepared in a complete medium, equal amounts of cells were seeded in 6-well plates (4 × 10^5^ cells/well) and then allowed for growth until being completely adherent. Subsequently, they were treated for different time periods (i.e., 0, 4, 16 h) with 50 μM of *t*BHQ. After collecting the cells in each group, they were resuspended in pre-cooled PBS (phosphate buffered saline). In order to determine intracellular ROS levels, all the experimental groups were exposed to 100 μM dichlofluorescein diacetate (DCFH-DA included in a detection kit, S0033S, Beyotime, Shanghai, China) for 30 min at the incubator (37 °C, 5% CO_2_). After being washed twice with PBS, all they were centrifuged and resuspended in serum-free media. The resulting 2′7′-dichlofluorescein (DCFH) was detected at the excitation wavelength of 488 nm and the emission wavelength of 525 nm by a flow cytometry (FlowJo, Ashland, OR, USA). The final results were expressed by the fluorescence intensity of DCFH detected in distinct cell lines. Furthermore, all the experimental cells were treated as abovementioned method and collected by centrifuging at 1000× *g* for 5 min, and stained with a binding buffer containing of both Annexin V-FITC and propidium iodide (PI) for 15 min. After the cells were washed twice to remove the excess staining reagent, they were subjected to detection of cell apoptosis by flow cytometry. The resulting data were shown by different fluorescence intensity in distinct states of cells.

### 2.6. The Assays for Total, Reduced, and Oxidized Glutathione Levels

All experimental cell lines were suspended in a complete medium, and then equal amounts of cells were allowed for growth in 6-well plates (4 × 10^5^ cells/well) until being completely adherent. Thereafter, they were treated for different time periods (i.e., 0, 4, 16 h) with 50 μM of *t*BHQ. All the cells were collected in PBS and then subjected to the measurement of total glutathione, reduced glutathione (GSH) and oxidized glutathione (GSSG) by using a glutathione assay kit (A061-1, Nanjing Jiancheng, Nanjing, China) according to the manufacturer’s instruction. Of note, two standards of GSH and GSSG were also prepared in the same assays. The assay was designed by employing an Ellman’s reagent (5,5’-disulfidebis-2-nitrobenzoic acid, DNTB), which can react with GSH to form 2-nitro-5-thiobenzoic acid, a yellow product with an absorbance at a wavelength of 405 nm. In addition, the protein concentrations in all experiment cells were determined by the bicinchoninic acid assay (BCA, P1511, ApplyGene Co., Beijing, China) and used as an internal control for the normalization, along with relevant standard curves, in order to calculate amounts of total glutathione, GSSG, and GSH by the formula provided by this manufacturer. The final resulting data are shown by a ratio of GSSG to GSH levels.

### 2.7. Assays for ROS-Scavenging Activities of Superoxide Dismutase and Catalase

Equal amounts of each of experimental cell lines suspended in a complete medium were seeded in 6-well plates (4 × 10^5^ cells/well) and then allowed for growth until being completely adherent. Thereafter, they were treated for different time periods (i.e., 0, 4, 16 h) with 50 μM of *t*BHQ. All groups of experimental cells had been collected and subjected to assays for ROS-scavenging activities of superoxide dismutase (SOD), that were determined according to the instruction of enhanced SOD assay kit (A001-3, Nanjing Jiancheng, Nanjing, China). Besides, another ROS-scavenging enzyme catalase (CAT) activity was detected through the instructions of CAT kit (BC0205, Solarbio, Beijing, China).

### 2.8. ARE-Luciferase Reporter Assays

All experimental cells were, separately, seeded into 12-well plates (1.5 × 10^5^ cells/well), and allowed for growth to reach 80% of confluence, before the cells were co-transfected using a lipofectamine 3000 mixture with each of ARE (antioxidant response elements)-driven luciferase plasmids (which were made by inserting each of the indicated ARE sequences into the pGL3-Promoter vector) or non-ARE reporter plasmids (as an internal background control), together with an experiment construct for Nrf1, Nrf2, or an empty pcDNA3.1 vector. In this test, the *Renilla* expression by pRL-TK (a plasmid encoding renilla luciferase driven by the thymidine kinase promoter) served as an internal quality control for transfection efficiency. Thereafter, the luciferase activity was measured by the dual-luciferase reporter system (Beyotime, Shanghai, China). The resulting data were calculated as fold changes (mean ± SD, *n* = 3 × 3) relative to the controls, which are representative of at least three independent experiments being each performed in triplicates.

### 2.9. Statistical Analysis

All the relevant results are presented as mean ± SD (*n* = 3 × 3 or 5 × 3) relative to the indicated controls. The comparison of the various experimental groups and their corresponding controls was carried out by one- way ANOVA, and analyzed by the post-hoc test with Fisher’s least significant difference (LSD). The differences in between distinct treatments were considered to be statistically significant at *p* < 0.05.

## 3. Results

### 3.1. Different Effects of Nrf1 and Nrf2 on Cell Growth during tBHQ Intervention

**Figure 1:**
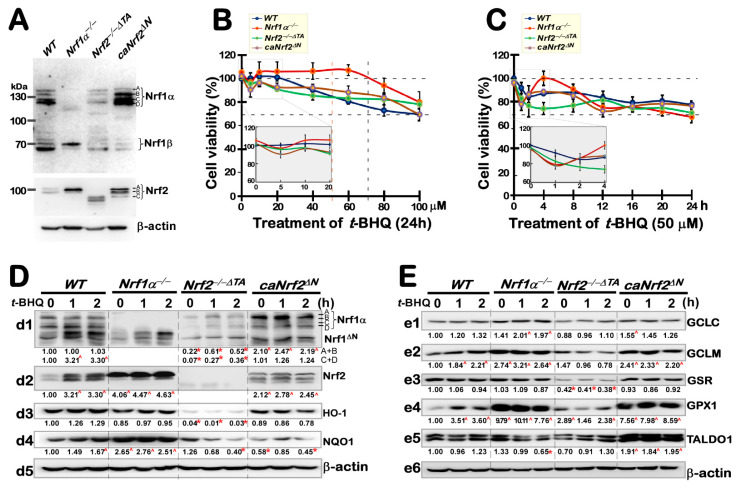
Distinct effects of *t*BHQ on different cell viability and antioxidant responsive genes. (**A**) Distinct protein abundances of Nrf1 and Nrf2 isoforms in *WT*, *Nrf1α*^−/−^, *Nrf2*^−/−^*^ΔTA^*, or *caNrf2^ΔN^* cell lines were determined by Western blotting with their specific antibodies. (**B**,**C**) Different cell viability was examined by the MTT-based assay, after the indicated cells had been treated with *t*BHQ: (**B**) at different doses (from 0 to 100 μM) for 24 h; (**C**) at a single dose of 50 μM for different lengths of time. The MTT assay was repeated three times, each performed in at least quintuplicates. The expanded graphic areas (at 0 to 20 μM between 0 and 4 h) were placed in the interior windows. (**D**,**E**) Four distinct cell lines were or were not treated with 50 μM *t*BHQ for a short time (from 0 to 2 h), followed by western blotting of Nrf1 (*d1*), Nrf2 (*d2*) and other ARE-driven target gene products as indicated (*d3*, *d4* and *e1–e5*). The intensity of immunoblots was calculated at the gray values listed under the corresponding protein bands with statistical analysis of significant increases (^, *p* < 0.01) and significant decreases (*, *p* < 0.01) in their expression levels. These data shown herein are representative of at least three independent experiments.

To gain an insight into endogenous Nrf1- and Nrf2-mediated antioxidant responses to *t*BHQ, we here confirmed that four distinct genotypes of cell lines are true (Figure 1A), as described previously [31,40]. Of striking note, human Nrf1α is manifested with four major isoforms, as identified by Xiang et al. [41], of which its A and B isoforms represent the full-length glycoprotein and deglycoprotein of Nrf1, respectively, whilst its C and D isoforms denote two distinct lengths of Nrf1′s N-terminally-truncated isoforms. Specific knockout of *Nrf1α* (by its gene-editing to delete a very short segment adjoining its translational start codons) led to a complete loss of all four *Nrf1α*-derived isoforms in *Nrf1α*^−/−^ cells, albeit with a retention of other two minor proteins Nrf1^ΔN^ and Nrf1β (Figure 1A). By contrast, all four Nrf1α-derived isoforms A to D were also substantially diminished by *Nrf2^−/−ΔTA^*, but its B to D isoforms (with distinct potentials of its *trans*-activity) were significantly augmented by *caNrf2^ΔN^*. Intriguingly, Nrf1β abundances were also markedly suppressed by *Nrf2^−/−ΔTA^* or *caNrf2^ΔN^*. These imply that Nrf1 expression and processing may be monitored by Nrf2, besides itself. Conversely, only a major Nrf2 isoform-A, but not its isoforms B or C, was incremented in *Nrf1α*^−/−^ cells (Figure 1A). However, all three isoforms A to C of Nrf2 were completely abolished by specific deletion of its transactivation Neh4-Neh5 domains (to yield an inactive *Nrf2^−/−ΔTA^* mutant), but their disappearance seemed to be replaced by other three smaller isoforms with a faster electrophoretic mobility (which were generated from Nrf2^ΔTAD^, a dominant-negative mutant retaining its prototypic DNA-binding activity competitively against relevant wild-type factors), when compared with those equivalents examined in *WT* cells. By contrast, three slightly shorter isoforms of *caNrf2^ΔN^* (closely to wild-type isoforms A to C, respectively) were retained and enhanced by this constitutive active mutant factor, because the N-terminal Keap1-binding Neh2 domain of Nrf2 was removed from its genomic locus. Collectively, these indicate that Nrf2 expression and processing may also be monitored by itself, as well as by Nrf1, within an inter-regulatory feedback cycle.

Next, the cytotoxic effect of *t*BHQ on the aforementioned four different cell lines was evaluated by a MTT assay for the formation of formazan precipitates with succinate dehydrogenase in the mitochondria of all living cells only, and changes in the absorbance were measured to reflect the cell viability. As shown in Figure 1B, the viability of three examined cell lines except wild-type (*WT*) cells was modestly decreased by intervention with 5 μM *t*BHQ, but 10 μM of this chemical enabled these cell viability to return closely to their basal levels (obtained from treatment of cells with the vehicle of 0.1% DMSO). Then, a relatively stable viability of *Nrf1α*^−/−^ cells was maintained between 10–60 μM of *t*BHQ, followed by a gradual decrease to 80% of its viability until its concentration increased to 100 μM (Figure 1B). By contrast, a narrow window of stable *WT* cell viability was defined by 5–20 μM *t*BHQ, followed by a fairly sloping downhill to 70% viability of the cells treated with 100 μM *t*BHQ, while the other two close smoothly growth curves emerged from 10 to 80 μM *t*BHQ treatments of either *Nrf2^−/−ΔTA^* or *caNrf2^ΔN^* cell lines, before their viability decreased to 80% and 70%, respectively, upon treatment of 100 μM *t*BHQ (Figure 1B).

Based on the dose-dependent effects, 50 μM *t*BHQ was selected for intervention of the above-described four cell lines to assess distinct time-dependent growth courses (Figure 1C). The results showed that the viability of all four cell lines decreased to different extents of between 90% and 75% by *t*BHQ intervention for 1 h. Of note, the continuous treatment enabled the viability of *WT* and *Nrf2^−/−ΔTA^* cell lines to smoothly decrease to 85–80% or 75–75% from 2 h or 4 h to 24 h, respectively (Figure 1C). By sharp contrast, the viability of *Nrf1α*^−/−^ and *caNrf2^ΔN^* cell lines appeared to elevate respectively to 100% or 90% in a modest ‘bounce-back’ response to *t*BHQ-continued treatment from 2 h to 4 h, and then both declined to 75% at 12 h of treatment. Thereafter, the viability of *Nrf1α*^−/−^ cells continued to gradually reduce to 70% until 24 h of *t*BHQ treatment, whilst the viability of *caNrf2^ΔN^* cells was maintained to 75% from 12 h to 24 h treatments. As such, all cell viability reached a relatively stable level of them after 16 h of *t*-BHQ intervention. Therefore, the optimal concentration of *t*BHQ and its optimal time course were selected in the follow-up experiments to assess the cytoprotective roles of *Nrf1* and *Nrf2* against this chemical. For this end, we mainly investigated their expressional differences between these four cell lines in responses to 50 μM *t*BHQ intervention for different time periods as indicated.

### 3.2. Short-Term Intervening Effects of tBHQ on Nrf1, Nrf2, and AREs-Driven Genes in Distinct Genotypic Cells

Herein, short-term effects of *t*BHQ intervention for 1–2 h on Nrf1 and Nrf2 were first examined by western blotting (Figure 1D,E). The results showed that *t*BHQ treatment of *WT* cells caused modest increases in Nrf1-processed isoforms C/D, as well as Nrf1^ΔN^ (Figure 1D (*d1*)). Such altered Nrf1^ΔN^ also emerged in *Nrf1α*^−/−^ cells, albeit it lacked A to D isoforms, implying it is not originated from the full-length Nrf1α processing. By contrast, a slight enhancement in the remnant Nrf1α-derived isoforms and Nrf1^ΔN^ expression in *t*BHQ-treated *Nrf2^−/−ΔTA^* cells, but both their basal and *t*BHQ-stimulated levels were increased in *caNrf2^ΔN^* cells (Figure 1D (*d1*)). For Nrf2, its protein expression was more sensitive to *t*BHQ stimulation in *WT* cells, and also increased significantly after 1 h treatment (Figure 1D (*d2*)), when compared with those in the other three cell lines. They appeared to be largely insensitive to *t*BHQ, even although altered Nrf2 expression levels were evidently enhanced in *Nrf1α*^−/−^ and *caNrf2^ΔN^*, except *Nrf2^−/−ΔTA^*, cells lines, but all three with no obvious changes after treatment with *t*BHQ.

Both basal and *t*BHQ-stimulated expression levels of ARE-driven genes regulated by Nrf1 and/or Nrf2 were determined next (Figure 1D,E). The results revealed that distinct expression levels of NQO1 (NAD(P)H:quinone oxidoreductase 1; Figure 1D (*d4*)), GCLM (glutamate-cysteine ligase modifier subunit; Figure 1E (*e2*)), GPX1 (glutathione peroxidase 1; Figure 1E (*e4*)), and HO-1 (heme oxygenase 1, also called HMOX1; Figure 1E (*d3*)) in *WT* cells were induced by *t*BHQ; this appeared to be accompanied by Nrf2 inducible enhancement. However, all these examined proteins and also others including GCLC (glutamate-cysteine ligase catalytic subunit; Figure 1E (*e1*)), GSR (glutathione-disulfide reductase, Figure 1E (*e3*) and TALDO (transaldolase 1, Figure 1E (*e5*), were largely unaffected by short-term *t*BHQ intervention of *Nrf1α*^−/−^ cells, even though basal abundances of NQO1, GCLM, and GPX1, amongst them aforementioned, were highly augmented as accompanied by hyper-expression of Nrf2. Similarly, constitutive active *caNrf2^ΔN^* also resulted in basal increases in GCLM, GPX1, and TALDO (Figure 1E (*e2*, *e4*, *e5*)), but as accompanied by a basal decrease of NQO1, whereas all these examined protein levels were almost unaltered by *t*BHQ stimulation of *caNrf2^ΔN^* cells. Conversely, knockout of *Nrf2^−/−ΔTA^* only led to reduced basal levels of both HO-1 and GSR (Figure 1D (*d3*) and Figure 1E (*e3*)), whilst *t*BHQ stimulation merely caused an inducible increase of TALDO alone in *Nrf2^−/−ΔTA^* cells (Figure 1E (*e5*)). Intriguingly, *t*BHQ-triggered *Nrf2^−/−ΔTA^* cells also gave rise to modest decreases of NQO1, GCLM, and GPX1 (Figure 1D (*d4*) and Figure 1E (*e2*, *e4*)). Altogether, these indicate that Nrf1 and Nrf2 could make differential yet integral contributions to basal and *t*BHQ-inducible expression levels of these examined ARE-driven genes. For further insights into differential expression patterns of these antioxidant cytoprotective genes among different genotypic cell lines, the following experiments were performed by long-term stimulation of cells with *t*BHQ for 4 h to 24 h.

### 3.3. Long-Term Stimulating Effects of tBHQ on Nrf1, Nrf2, and Downstream Targets in Distinct Genotypic Cells

**Figure 2:**
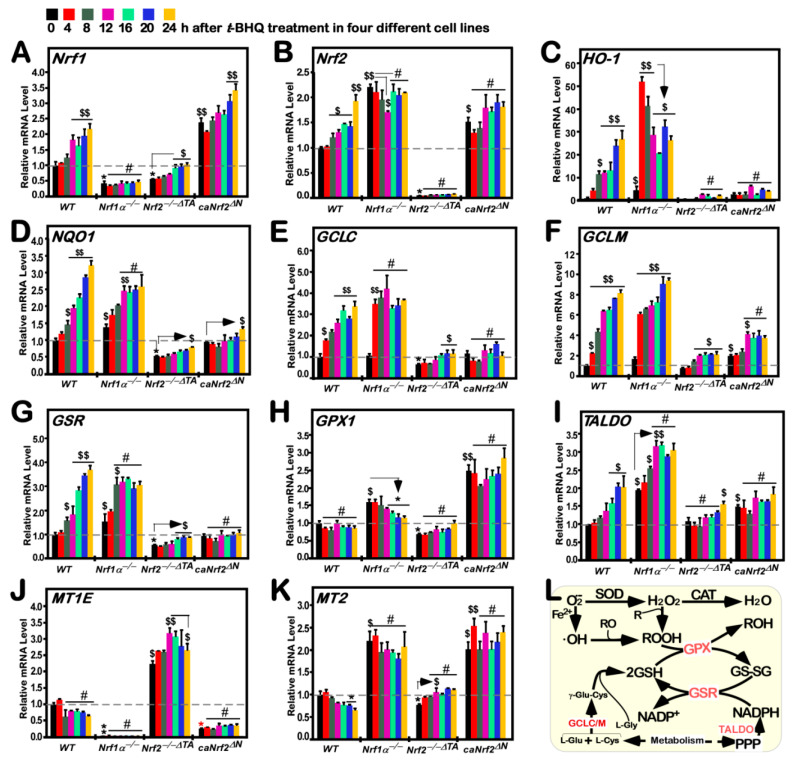
Time-dependent changes in the mRNA expression of distinctive responsive genes to tBHQ. Distinct genotypic cell lines of *WT*, *Nrf1α*^−/−^, *Nrf2*^−/−^*^ΔTA^* or *caNrf2^ΔN^* were (or were not) treated with 50 μM *t*BHQ for 0 to 24 h, before both basal and *t*BHQ-inducible mRNA expression levels of all examined genes were determined by RT-qPCR. (**A**–**K**) These genes included *Nrf1* (**A**), *Nrf2* (**B**), *HO-1* (**C**), *NQO1* (**D**), *GCLC* (**E**), *GCLM* (**F**), *GSR* (**G**), *GPX1* (**H**), *TALDO* (**I**), *MT1E* (**J**), and *MT2* (**K**). Then, mRNA expression was calculated as described in “Section 2”. The resulting data were shown as fold changes (mean ± SD, *n* = 3 × 3), which are representative of at least three independent experiments being each performed in triplicates. Significant increases ($, *p* < 0.05; $$, *p* < 0.01) and significant decreases (* p < 0.05; ** *p* < 0.01), in addition to the non-significance (#), were statistically analyzed when compared with the corresponding cell line controls (measured at 0 h), respectively. (**L**) A schematic representation of several major ROS-scavenging enzymes (e.g., SOD and CAT) and relevant redox signaling (e.g., GPX and GSR) to defend against oxidative stress over-stimulated by ROS along with oxygen free radicals. Of note, as two important antioxidant players, GSH and NADPH are yielded from cell metabolism through key enzymes GCLC/M and TALDO, respectively. Thereby, these key gene products exert their vital redox-regulatory functions in antioxidant, detoxification, and cytoprotective processes.

To give a proper understanding of long-term *t*BHQ-stimulated effects on *Nrf1*, *Nrf2*, and downstream genes, their mRNA expression levels were determined by quantitative real-time PCR (Figure 2). The results revealed an obvious increase of *Nrf1* mRNA expression after 12-h *t*BHQ stimulation of *WT* cells; this increase was maintained to 24 h of treatment of this chemical (Figure 2A and Figure S1A). By contrast, basal mRNA expression level of *Nrf1* (measured at 0 h) was substantially abolished or diminished by knockout of *Nrf1α*^−/−^ or *Nrf2^−/−ΔTA^*, respectively. Therefore, although Nrf2 was rather highly expressed in *Nrf1α*^−/−^ cells (Figure 1D and Figure 2B), the remnant Nrf1 shorter isoforms in *Nrf1α*^−/−^ cells were insensitive to *t*BHQ (Figure 2A and Figure S1A), whereas the residual Nrf1 in *Nrf2^−/−ΔTA^* cells could also hardly trigger a marginal response to this chemical. Conversely, *caNrf2^ΔN^* cells had given rise to a remarkable increase in basal *Nrf1* mRNA levels, but only a modest *t*BHQ-inducible increase of *Nrf1* expression was detected after 20–24 h stimulation of this cell line (Figure 2A and Figure S1A). Together, these results indicate that transcriptional expression of human *Nrf1* gene is monitored by Nrf2, as well by Nrf1 itself, even in the response to *t*BHQ.

Treatment of *WT* cells with *t*BHQ caused a gradual modest induction of *Nrf2* mRNA expression levels from 8 h to16 h, which was maintained to 20 h, followed by a marked peak of its induction at 24 h, of this chemical stimulation (Figure 2B and Figure S1B). Both basal and *t*BHQ-stimulated *Nrf2* expression levels were completely abolished by *Nrf2^−/−ΔTA^*. Rather, it is interesting that, even though basal *Nrf2* mRNA expression was significantly augmented by *Nrf1α*^−/−^ or *caNrf2^ΔN^*, its *t*BHQ-stimulated expression levels were roughly unaffected or even partially reduced in such two distinct genotypic cell lines (Figure 2B and Figure S1B). Collectively, these demonstrate that transcriptional expression of human *Nrf2* gene is bidirectionally regulated by itself and Nrf1, but upon stimulation by *t*BHQ, itself regulation by Nrf2 per se appears to be attributable to its N-terminal Keap1-binding Neh2 domain of the latter CNC-bZIP (Cap’n’Collar basic region-leucine zipper) factor.

Besides Nrf1 and Nrf2, downstream target genes *HO*-*1* (Figure 2C and Figure S1C) and *NQO1* (Figure 2D and Figure S1D) were also induced by *t*BHQ treatment of *WT* cells in a time-dependent manner. Upon loss of Nrf1α-derived isoforms, significant increments in basal and *t*BHQ-stimulated mRNA expression levels of *HO*-*1* and *NQO1* were determined in *Nrf1α*^−/−^ cells. The first sharp maximum peak of *HO*-*1* occurred at 4 h of induction by *t*BHQ, followed by a gradual decline to 16 h and then the second peak at 20 h treatment of *Nrf1α*^−/−^ cells (Figure 2C and Figure S1C). By contrast, only a smooth increase of *NQO1* was obtained from 4 h to 12 h of *t*BHQ induction of *Nrf1α*^−/−^ cells to a higher level, which was then maintained at such high level until 24 h (Figure 2D and Figure S1D). However, loss of *Nrf2^−/−ΔTA^* led to an evident diminishment or even abolishment in basal and *t*BHQ-stimulated expression levels of *HO*-*1* and *NQO1* (Figure 2C,D), except for a marginal induction of *NQO1* by *t*BHQ at 24 h. Conversely, constitutive active *caNrf2^ΔN^* appeared to have no significant effects on both basal and *t*BHQ-stimulated expression of *HO*-*1* and *NQO1* (Figure 2C,D)*,* albeit with a weak induction of *NQO1* by *t*BHQ at 24 h and another similar lower stimulation of *HO*-*1* at 12 h to 20 h (Figure S1C,D). Altogether, these data indicate that *HO*-*1* and *NQO1* serve as two representative targets of Nrf2, and both genes regulated by Nrf2 may also be monitored positively by its N-terminal Neh2 domain, aside from the potential negative regulation of Nrf2 and its targets by Nrf1.

### 3.4. Long-Term Stimulation of Human Antioxidant and Detoxification Genes by tBHQ in Distinct Genotypic Cells

It is of crucial antioxidant and detoxification to be merited by glutathione (GSH)-conjugates in the redox signaling cycles. The intracellular biosynthesis of GSH, as an important cellular antioxidant, is controlled by a key rate-limiting enzyme consisting of both GCLC and GCLM subunits. As shown in Figure 2E,F, a time-dependent increment in the mRNA expression of *GCLC* and *GCLM* induced by *t*BHQ from 4 h to 24 h was determined in *WT* cells (also see Figure S1E,F). By contrast, *Nrf1α*^−/−^ cells gave rise to a rapid induction of *GCLC* mRNA expression by 4-h of *t*BHQ stimulation, to a maximum peak similar to that of *WT* cells, which was then maintained at a higher level until 24 h (Figure 2E and Figure S1E). However, no striking changes in both basal and *t*BHQ-stimulated *GCLC* expression were observed in *Nrf2^−/−ΔTA^* or *caNrf2^ΔN^* cell lines, albeit with a few exceptions of marginal alternations (Figure S1E). Interestingly, further examinations revealed that basal and *t*BHQ-stimulated *GCLM* expression levels were substantially augmented in *Nrf1α*^−/−^ cells as the time was extended to 24 h treatment (Figure 2F and Figure S1F), whilst *caNrf2^ΔN^* cells only gave rise to a relatively lower induction of *GCLM* by *t*BHQ, although its basal expression was also certainly elevated at a similar level to that obtained from *Nrf1α*^−/−^ cells. Of note, *Nrf2^−/−ΔTA^* cells could still retain a considerably lower induction of *GCLM* by *t*BHQ to the constructive basal level of *caNrf2^ΔN^* (Figure 2F and Figure S1F). Altogether, these demonstrate differential contributions of Nrf1 and Nrf2 to basal and inducible regulation of both *GCLC* and *GCLM* genes controlling GSH biosynthesis in distinct genotypic cells.

As a central enzyme of the intracellular antioxidant defense, GSR can reduce the oxidized glutathione disulfide (GSSG) to the sulfhydryl form (GSH). In such a thiol-based redox cycle, another key enzyme GPX1, belonging to the glutathione peroxidase family, can catalyze the glutathione to reduce hydrogen peroxide (H_2_O_2_) and other organic hydroperoxides, in order to detoxify the oxidants and hence protect the cells from oxidative damages. Thereby, we examine the intervening effects of *t*BHQ on induction of *GSR* and *GPX1* mRNA expression mediated by Nrf1 and/or Nrf2 in distinct genotypic cell lines. As anticipated, RT-qPCR results revealed that *GSR* mRNA levels were strikingly gradually upregulated by *t*BHQ stimulation of *WT* cells from 8 h to 24 h (Figure 2G and Figure S1G), while *GPX1* expression was unaffected by this chemical treatment (Figure 2H and Figure S1H). Upon knockout of *Nrf1α*^−/−^, basal *GSR* and *GPX1* mRNA levels were markedly enhanced, but only modest induction of *GSR*, rather than GPX1, by *t*BHQ occurred from 4 h to 8 h and thereafter maintained at a maximum level that was yet lower than equivalent values measured from *t*BHQ-treated *WT* cells (Figure 2G,H). Such *t*BHQ-trigged induction of *GSR*, as well as its basal expression levels, was substantially attenuated or abolished by knockout of *Nrf2^−/−ΔTA^* (Figure 2G and Figure S1G). However, *GPX1* was smoothly downregulated by *t*BHQ stimulation of *Nrf1α*^−/−^ cells (Figure 2H and Figure S1H), though hyper-expression of Nrf2 was preserved in this knockout cell line (Figure 2B), but this effect appeared to be completely prevented by *Nrf2^−/−ΔTA^* (Figure 2H and Figure S1H). Conversely, constitutive active *caNrf2^ΔN^* only gave rise to a remarkable increase in basal expression levels of *GPX1*, but not *GSR*, except that both genes were almost insensitive to stimulation by *t*BHQ (Figure 2G,H).

Furthermore, TALDO is a key enzyme of the non-oxidative pentose phosphate pathway (PPP) providing ribose-5-phosphate for nucleic acid synthesis and NADPH for lipid biosynthesis [42]. Notably, NADPH arising from this pathway enables glutathione to be maintained at a reduced state, thereby protecting sulfhydryl groups and cellular integrity from oxygen radicals. Here, our results unraveled a stepwise inducible increase of *TALDO* mRNA expression levels from 4 h to 20 h of its maximum stimulation by *t*BHQ of *WT* cells, before being maintained until 24 h of this chemical stimulation (Figure 2I and Figure S1I). By contrast, *Nrf1α*^−/−^ caused significant increments in basal and *t*BHQ-stimulated expression levels of *TALDO* from 4 h to 12 h of its rapidly inducible peak that was much higher than the values obtained from *WT* control cells, which was then largely retained to 24 h. Such induction of *TALDO* was almost abolished by *Nrf2^−/−ΔTA^* (except for a marginal induction of it by 24-h stimulation of *t*BHQ), and also suppressed by *caNrf2^ΔN^* (albeit its basal levels were augmented) (Figure 2I and Figure S1I). Collectively, these indicate that Nrf1 and Nrf2 exert differential yet integral roles in mediating the aforementioned antioxidant cytoprotective genes against *t*BHQ.

In addition, it is worth mentioning that metallothioneins (MT) cannot only maintain the metal homeostasis in vivo, but also serve as a redox buffer for ROS and other free radicals to play an essential role in the cytoprotective process [43]. However, our examinations of *MT1E* and *MT2* unraveled that both genes were not merely insensitive to *t*BHQ, but were modestly downregulated by this chemical intervention of *WT* cells (Figure 2J,K, and see Figure S1J,K). Of note, basal mRNA expression of *MT1E*, along with its inhibitory effect of *t*BHQ, was markedly diminished or completely abolished in *caNrf2^ΔN^* or *Nrf1α*^−/−^ cells, respectively (Figure 2J and Figure S1J), but both cell lines gave rise to a remarkable increase of basal *MT2* expression, aside from that *t*BHQ-stimulated expression of *MT2* was elevated in *caNrf2^ΔN^*, rather than *Nrf1α*^−/−^, cells (Figure 2K and Figure S1K). By striking contrast, *Nrf2^−/−ΔTA^* cells could give rise to significant increases in basal and *t*BHQ-inducible mRNA expression profiles of *MT1E* from 4 h to 12 h of this chemical stimulation, prior to being maintained at a considerably higher levels until 24 h (Figure 2J and Figure S1J), whereas basal *MT2* expression level was modestly downregulated by *Nrf2^−/−ΔTA^*, but with a marginal induction by *t*BHQ treatment from 12 h to 24 h (Figure 2K and Figure S1K). Together, these suggest a remarkable distinction in contributions of Nrf1 and Nrf2 to transcriptional regulation of *MT1E* and *MT2*, respectively.

### 3.5. Distinct Time-Dependent Effects of tBHQ on Nrf1, Nrf2, and Target Gene Expression in Different Cell Lines

**Figure 3:**
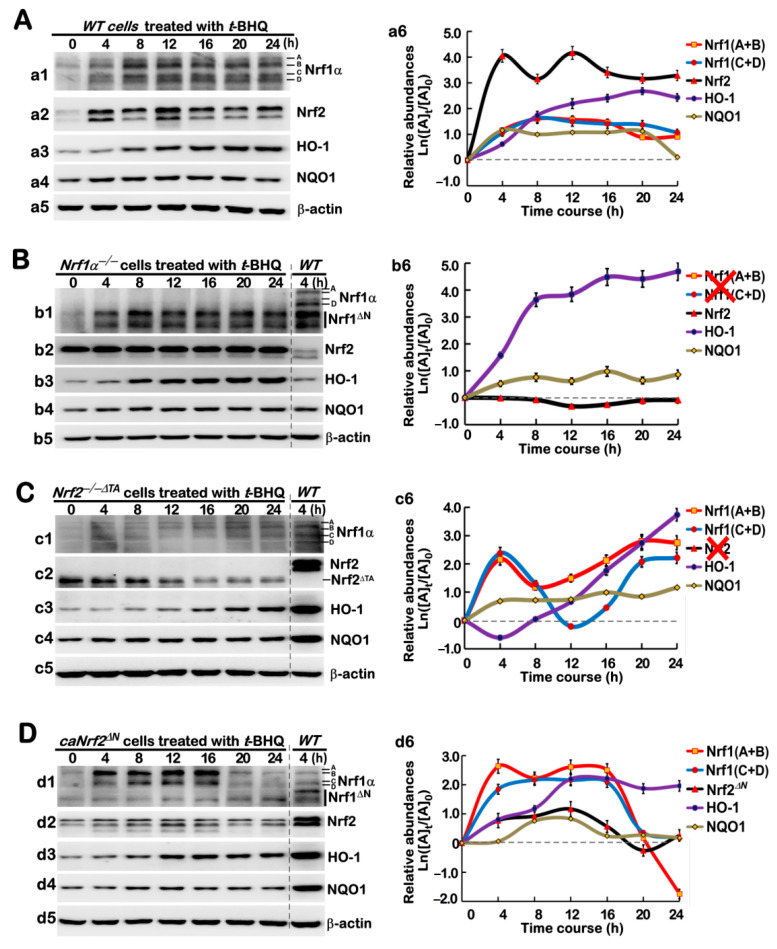
Long-term effects of *t*BHQ on protein abundances of Nrf1, Nrf2 and co-target genes. Experimental cells, *WT* (**A**), *Nrf1α*^−/−^ (**B**), *Nrf2*^−/−^*^ΔTA^* (**C**), and *caNrf2^ΔN^* (**D**), were or were not treated with 50 μM *t*BHQ for 0 to 24 h, before basal and *t*BHQ-inducible protein changes of Nrf1 (*a1*, *b1*, *c1*, *d1*), Nrf2 (*a2*, *b2*, *c2*, *d2*), HO-1 (*a3*, *b3*, *c3*, *d3*), and NQO1 (*a4*, *b4*, *c4*, *d4*) were determined by Western blotting with their indicated antibodies, whilst β-actin served as a loading control. The intensity of those immunoblots, representing different protein expression levels, was also quantified by the Quantity One 4.5.2 software (Bio-Rad, Hercules, CA, USA). The data were representative of at least three independent experiments, as shown graphically (in right panels), after being calculated by a formula of Ln([A]_t_/[A]_0_, in which [A]_t_ indicated a fold change (mean ± SD) in each of those examined protein expression levels at different times relative to the corresponding controls measured at 0 h (i.e., [A]_0_). In addition, it should be noted that two big red crosses represent the constitutive losses of *Nrf1* or *Nrf2* (*b6*, *c6*), respectively.

As shown in Figure 3A (*a1*), Nrf1α-derived isoforms A to D were obviously enhanced after 4 h of *t*BHQ treatment in *WT* cells and then maintained to their considerably higher extents between 8 h and 24 h, as illustrated graphically (Figure 3A (*a6*). Similarly, the abundance of Nrf2 proteins was rapidly significantly augmented by *t*BHQ stimulation of *WT* cells from 1 h to 24 h (Figure 1D (*d2*) and Figure 3A (*a2*)). Although hyper-expressed Nrf2 was retained in *Nrf1α*^−/−^ cells, its protein abundances were unaffected by *t*BHQ intervention of Nrf1α-specific knockout cells (Figure 3B (*b2*, *b6*)), in which Nrf1α-derived isoforms were constitutively lacked, but its N-terminal portion-truncated Nrf1^ΔN^ abundances were rather promoted by *t*BHQ (Figure 3B (*b1*)). By contrast, *Nrf2^−/−ΔTA^* cells could only yield considerably weaker abundances of Nrf1α-derived isoforms A to D, but they still were enabled to respond to *t*BHQ in a biphasic manner, with the first peak at 4 h of this stimulation and the recurring second peak at 20 h of stimulation (Figure 3C (*c1*, *c6*)), whilst the remnant Nrf2^ΔTAD^ mutant proteins were evidently time-dependently inhibited by *t*BHQ intervention of *Nrf2^−/−ΔTA^* cells (Figure 3C (*c2*)), as compared with the positive reference control of 4-h *t*BHQ-treated *WT* cells (as indicated in the same gels). Conversely, *caNrf2^ΔN^* cells led to strikingly increased abundances of basal Nrf2^ΔN^ and Nrf1α-derived isoforms (Figure 1D (*d1*, *d2*)), and also their time-dependent inducible expression changes were here determined in *t*BHQ-stimulated *caNrf2^ΔN^* cells (Figure 3D (*d1*, *d2*, *d6*)).

Next, further examinations revealed that *t*BHQ-inducible expression levels of HO-1 in *WT* cells were gradually incremented, as its intervening time extended from 4 h to 20 h, to a considerably higher level, before being slightly declined (Figure 3A (*a3*, *a6*)), whilst NQO1-induced expression levels were rapidly triggered by 4-h of this stimulation to a certain extent, and then maintained until 24 h (Figure 3A (*a4*, *a6*)). Of great note, although basal abundance of HO-1 was substantially diminished by *Nrf2^−/−ΔTA^* (Figure 1D (*d3*)), it remained to be significantly induced by *t*BHQ from 8 h to 24 h of stimulation to a maximum extent (Figure 3C (*c3*, *c6*)), whilst another rapid modest induction of NQO1 by *t*BHQ were detected in *Nrf2^−/−ΔTA^* cells (Figure 3C (*c4*, *c6*)), when they compared to a positive reference control obtained from 4-h *t*BHQ-treated *WT* cells (as shown in the same gels). In *Nrf1α*^−/−^ cells, even though the hyper-expressed Nrf2 was insensitive to *t*BHQ, both HO-1 and NOQ1 were still rapidly induced by this chemical from 4 h to 8 h of stimulation and then maintained to their respective higher extents until 24 h (Figure 3B (*b3*, *b4*,*b6*)). Rather, only a marginal induction of *caNrf2^ΔN^* by *t*BHQ was observed, but this was accompanied by significant induction of HO-1 and NQO1 (but still were lower than the positive control level of 4-h *t*BHQ-treated *WT* cells), as well as Nrf1α-derived proteins (to a greater extent than the *t*BHQ-treated *WT* controls), which occurred in their distinct time-dependent courses (Figure 3D (*d1*–*d6*)). Taken together, these results demonstrate that such two inter-regulatory factors of Nrf1 and Nrf2—together with distinct targets—could mediate differential yet integral responses to *t*BHQ intervention of different genotypic cell lines.

### 3.6. Different Time-Dependent Effects of tBHQ on Antioxidant Cytoprotective Gene Expression in Distinct Cell Lines

**Figure 4:**
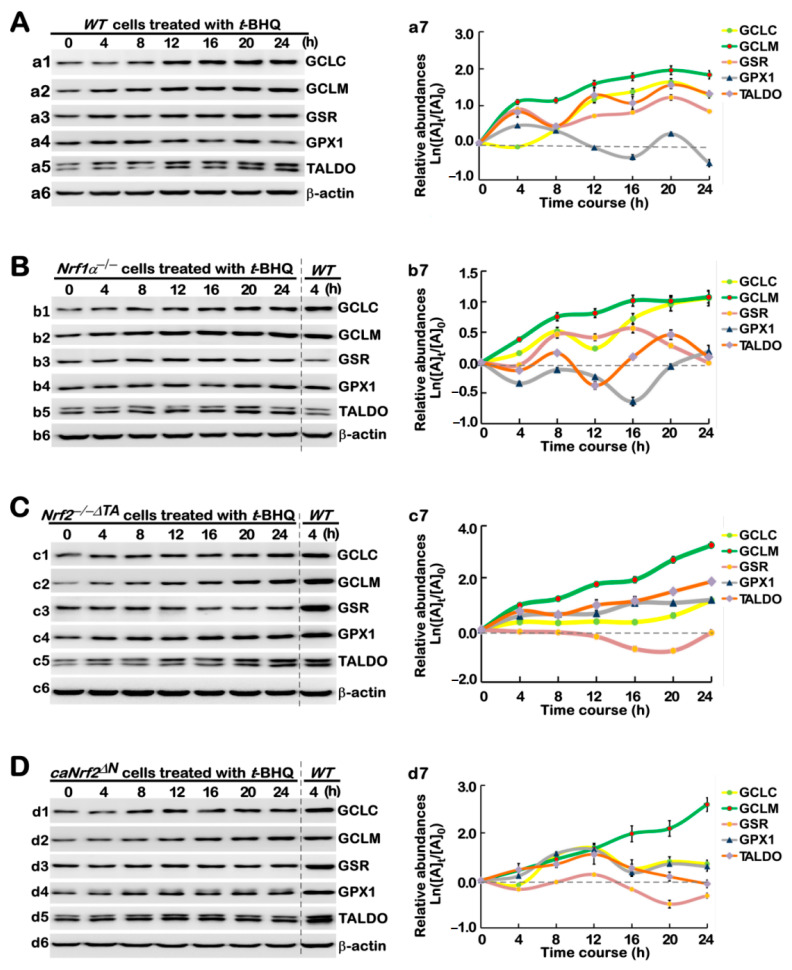
Long-term *t*BHQ-stimulated changes in the protein expression of antioxidant responsive genes. Different lines of *WT* (**A**), *Nrf1α*^−/−^ (**B**), *Nrf2*^−/−^*^ΔTA^* (**C**), and *caNrf2^ΔN^* (**D**) were treated with 50 μM *t*BHQ or not for 0 to 24 h, before basal and stimulated abundances of those antioxidant cytoprotective proteins, e.g., GCLC (*a1*, *b1*, *c1*, *d1*), GCLM (*a2*, *b2*, *c2*, *d2*), GSR (*a3*, *b3*, *c3*, *d3*), GPX1 (*a4*, *b4*, *c4*, *d4*), and TALDO (*a5*, *b5*, *c5*, *d5*), were determined by western blotting with the indicated antibodies. The intensity of relevant immunoblots representing different protein expression levels was also quantified by the Quantity One 4.5.2 software. The resulting data were then shown graphically (in right panels), after being calculated by a formula of Ln([A]_t_/[A]_0_), in which [A]_t_ indicated a fold change (mean ± SD) in each of those examined protein expression levels at different times relative to the corresponding controls measured at 0 h (i.e., [A]_0_), which were representative of at least three independent experiments.

As shown in Figure 4A, *t*BHQ stimulation of *WT* cells caused significant time-dependent induction of GCLC, GCLM, GSR, GPX1, and TALDO (*a1* to *a7*). Amongst them, GCLC was relatively slowly induced after 8 h of *t*BHQ stimulation and then gradually incremented to a maximum inducible extent at 20 h of this chemical treatment, before being slightly declined (*a1*, *a7*). In contrast, GCLM, GSR, and TALDO was rapidly induced within 4 h of stimulation by *t*BHQ and then presented within stepwise ascending trends from 8 h to 24 h of this treatment (*a2*, *a3*, *a5* and *a7*), whilst GPX1 induction by *t*BHQ appeared to rise and fall within a biphasic waving mode (*a4*, *a7*). Such differences in these examined enzymes may be attributable to distinct involvement of their upstream factors (e.g., Nrf1 and Nrf2) in the cellular response to *t*BHQ.

Next, we determine distinct contributions of Nrf1 and Nrf2 to alterations in *t*BHQ-stimulated abundances of antioxidant and detoxification enzymes. As revealed in *Nrf1α*^−/−^ cells, GCLC and GCLM were successively induced by *t*BHQ from 4 h to 24 h of their maximum stimulation (Figure 4B, (*b1*, *b2*, *b7*)), while a lag induction of GSR occurred at 8 h of *t*BHQ stimulation, which was maintained to 16 h and then declined gradually to its basal levels at 24 h of this treatment (*b3* and *b7*). As such, TALDO only displayed modest induction by *t*BHQ in a biphasic stepwise, with the first induction at 8 h and the second higher induction at 20 h before be declined nearly to its basal level (*b5* and *b7*), except largely no induction of GPX1 in *Nrf1α*^−/−^ cells (*b4* and *b7*), although hyper-active Nrf2 was retained. However, the inactive *Nrf2^−/−ΔTA^* could still give rise to gradual enhancements in inducible GCLC, GCLM, GPX1, and TALDO abundances from 4 h to 24 h of *t*BHQ stimulation (Figure 4C (*c1*, *c2*, *c4*–*c7*)), albeit all occurred to lower extents than the positive control levels of 4-h *t*BHQ-treated *WT* cells (as in the same gels), whereas GSR was slightly downregulated by this chemical in *Nrf2^−/−ΔTA^* cells (*c3* and *c7*). By sharp contrast, *caNrf2^ΔN^* could also only lead to a significant increment in induction of GCLM by *t*BHQ from 4 h to 24 h (Figure 4D (*d2*, *d7*)), in addition to only modest induction of GCLC, GPX1,TALDO, but not GSR, by this chemical stimulation, which was maintained from 8 h to 12 h and then declined to relatively lower levels (*d1*, *d3*–*d7*).

### 3.7. Different Antioxidant Responses of Four Distinct Genotypic Cell Lines to tBHQ as a Pro-Oxidative Stressor

**Figure 5:**
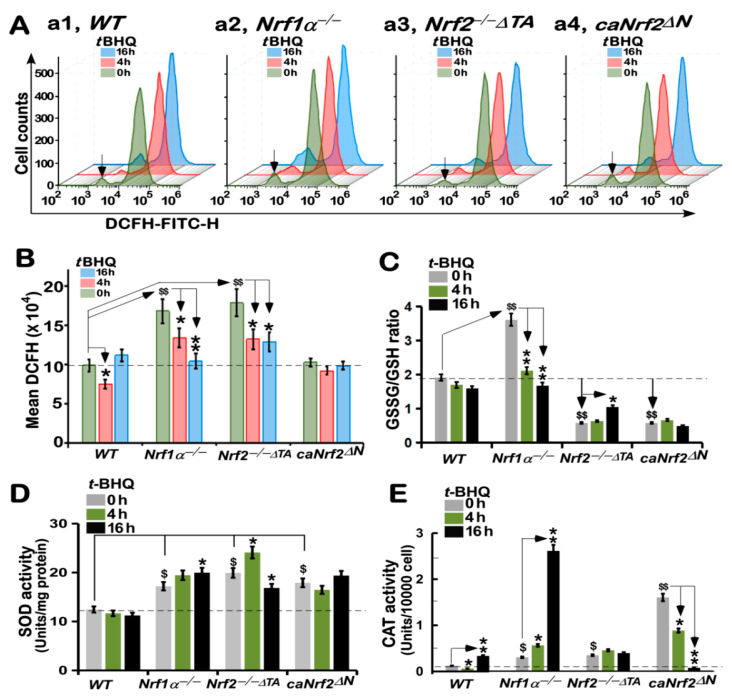
Altered levels of ROS, GSSG and GSH, along with ROS-scavenging activity of SOD and CAT, induced by *t*BHQ. (**A**) Experimental cells of *WT*, *Nrf1α*^−/−^, *Nrf2^−/−ΔTA^*, and *caNrf2^ΔN^* were allowed for treatment with 50 μM *t*BHQ or not for different time periods (i.e., 0, 4, 16 h). Thereafter, the cells were subjected to a flow cytometry analysis of intracellular ROS by the DCFH-DA fluorescent intensity. The resulting data were further analyzed by FlowJo 7.6.1 software, as shown in the column charts (**B**). (**C**) The intracellular GSH and GSSH levels, together with two ROS-scavenging activities of SOD (**D**) and CAT (**E**), were measured according to the introduction of relevant kit manufacturers. All the experiment was repeated three times, each of which was performed in triplicates. Their statistic significances were determined as described in Section 2. Of note, $$, *p* <0.01, and $, *p* < 0.05 indicate significant differences calculated by comparing each basal value of [*Nrf1α*^−/−^]*_T0_*, [*Nrf2^−/−ΔTA^*]*_T0_*, and [*caNrf2^ΔN^*]*_T0_* with that of [*WT*]*_T0_*, while both * *p* < 0.05 and ** *p* < 0.01 denote significant differences of those values from each of cell lines treated by *t*BHQ for 4 h (i.e., [X]_T4_) or 16 h (i.e., [X]_T16_) versus its untreated [X]_T0_ value in the same group.

The above experiments revealed there exists a synergistic effect of those antioxidant and detoxification genes regulated by Nrf1 and/or Nrf2. Just such synergistic effects can fully ensure the stable and effective function of this antioxidant cytoprotective system (to yield GSH and NADPH) to remove the excessive ROS produced from oxidative stressor, so that a certain redox homeostasis is being maintained to ensure the proper physiological operation of a heathy body. As a general term, ROS represents a set of all oxygen-containing reactive substances, including superoxide anion, hydrogen peroxide, and relevant free radicals. To date, they remain to be hardly detected, owing to their characteristics of strong oxidative activity with such a short life to be rapidly scavenged and detoxified by antioxidants (i.e., GSH). As such, the intracellular redox state was herein measured directly by DCFH-DA, one of the most widely-used assays to evaluate the resulting oxidative damages, because it can react directly with ROS to give rise to an extremely sensitive, but impermeable, dichlofluorescein probe, as detected by flow cytometry [44]. As shown in Figure 5A, a left shift of the dichlofluorescein image resulted from 16-h *t*BHQ intervention of *WT* cells (*a1*, also see Figure S2), implying a relative decrease of intracellular ROS levels, when compared with the control image obtained from the untreatment with *t*BHQ (i.e., 0.1% DMSO vehicle at 0 h). Further examinations revealed that *Nrf1α*^−/−^ or *Nrf2^−/−ΔTA^* gave rise to a significant increase in basal ROS levels under the vehicle-treated conditions, and also an evident left-shift of their *t*BHQ-intervening images to varying extents (Figure 5A,B (*a2*, *a3*) and Figure S2), when compared with those measured from the *WT* cells. These indicate that, despite loss of Nrf1 or Nrf2 alone, the remaining portions of both factors still enabled either *Nrf1α*^−/−^ or *Nrf2^−/−ΔTA^* cell lines to be stimulated by *t*BHQ to trigger antioxidant cytoprotective responses against their endogenic oxidative stress. However, it is rather intriguing that almost no changes in both basal and *t*BHQ-stimulated dichlofluorescein images were determined in *caNrf2^ΔN^* cells, when compared to *WT* cells (Figure 5A,B (*a4*) and Figure S2). This implies that the N-terminal Keap1-binding domain of Nrf2 is required for mediating *t*BHQ-triggered antioxidant response.

Further glutathione assays unraveled that the ratio of GSSG to GSH was marginally reduced by *t*BHQ stimulation of *WT* cells (Figure 5C). By sharp contrast, *Nrf1α*^−/−^ led to a remarkable increase in its basal GSSG to GSH ratio, but significant decreases of this ratio occurred after *t*BHQ stimulation. This indicates putative endogenous oxidative stress to yield the excessive GSSG, more than GSH levels, in this Nrf2-hyperexpressed *Nrf1α*^−/−^ cells, but the remaining antioxidant response in this knockout cell line may be still triggered by *t*BHQ. Contrarily, *Nrf2^−/−ΔTA^* and *caNrf2^ΔN^* further caused substantial decreases in their basal GSSG to GSH ratio, although their stimulated ratios were less or not promoted by *t*BHQ, respectively (Figure 5C). This implicates such two distinctive mutants can still enable to yield a certain amount of GSH in *Nrf2^−/−ΔTA^* and *caNrf2^ΔN^* cell lines, but could not be enough to allow for effective conversion of GSH into GSSG, even under *t*BHQ-stimulated conditions.

To gain insights into the initial scavengers of ROS, the activity of two key enzymes—superoxide dismutase (SOD) and catalase (CAT)—was examined herein. As shown in Figure 5D, significant increases in the basal activity of SOD were determined in *Nrf1α*^−/−^, *Nrf2^−/−ΔTA^*, or *caNrf2^ΔN^* cell lines, and *t*BHQ-stimulated activity of SOD was further promoted only in *Nrf1α*^−/−^, *Nrf2^−/−ΔTA^*, but not *caNrf2^ΔN^*, cell lines. Of note, a longer term (of 16 h) treatment of *Nrf2^−/−ΔTA^* cells with *t*BHQ caused a substantial reduction of SOD activity to its basal level, which was, though, still higher than that measured from *WT* cells (Figure 5D). However, *caNrf2^ΔN^* cells displayed no significant changes in *t*BHQ-inducible SOD activity, albeit its basal activity was much more than that of *WT* cells. Additionally, no obvious changes in the SOD activity were detected in *WT* cells that had or had not been treated by *t*BHQ. However, further examinations revealed that CAT activity was evidently stimulated by *t*BHQ in *WT* cells (Figure 5E). By contrast, basal CAT activity was increased in both cell lines of *Nrf1α*^−/−^ and *Nrf2^−/−ΔTA^*, but its *t*BHQ-stimulated activity was markedly elevated only in *Nrf1α*^−/−^, rather than *Nrf2^−/−ΔTA^*, cells (Figure 5E). Furthermore, *t*BHQ stimulation of *caNrf2^ΔN^* cells caused a striking suppression or even complete abolishment of its inducible CAT activity at 4 h or 16 h of this treatment, respectively, although its basal activity was greatly substantially augmented. Such discrepant activities of SOD and CAT in between these cell lines, together with their differential expression results as published previously [45], demonstrate to be attributable to distinctive yet cooperative contributions of Nrf1 and Nrf2 at regulating different target genes.

### 3.8. Distinct Roles of Nrf1 and Nrf2 in Different Cell Apoptosis Induced by tBHQ as a Pro-Oxidative Stressor

**Figure 6:**
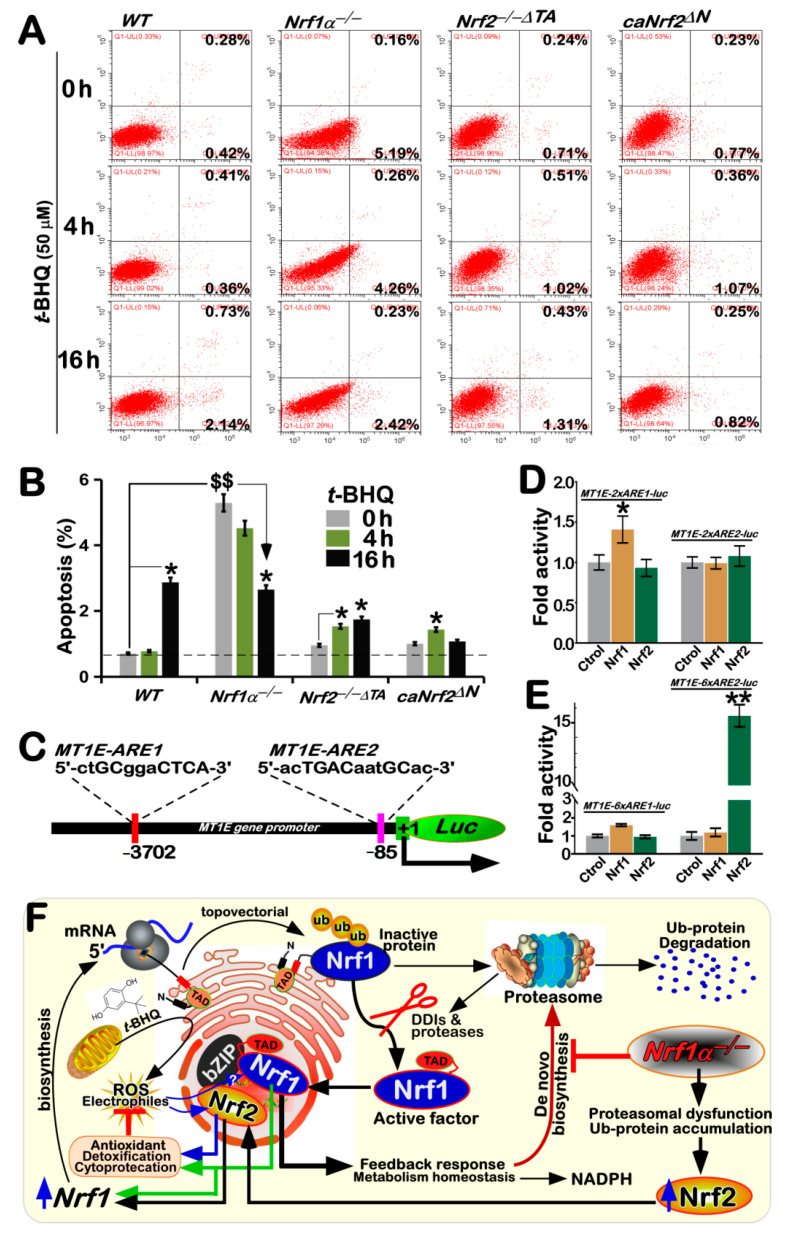
Nrf1 is more potent than Nrf2 at mediating the putative cytoprotective response to *t*-BHQ. (**A**) Distinct genotypic cell lines of *WT*, *Nrf1α*^−/−^, *Nrf2^−/−ΔTA^*, and *caNrf2^ΔN^* were or were not treated with 50 μM *t*BHQ for different lengths of time. Subsequently, the cells were incubated with a binding buffer containing Annexin V-FITC and propidium iodide (PI) for 15 min, before being subjected to the flow cytometry analysis of apoptosis. (**B**) The final results were shown by the column charts, which were representative of at least three independent experiments being each performed in triplicate. Of note, $$, *p* <0.01, indicate significant differences in each basal value of [*Nrf1α*^−/−^]*_T0_*, [*Nrf2^−/−ΔTA^*]*_T0_*, [*caNrf2^ΔN^*]*_T0_* versus that of [*WT*]*_T0_*, while both * *p* < 0.05 and ** *p* < 0.01 denote significant differences of those values from each of cell lines treated by *t*BHQ for 4 h (i.e., [X]_T4_) or 16 h (i.e., [X]_T16_) versus its untreated [X]_T0_ value in the same group. (**C**) Two putative ARE sequences were schematically shown in the location of the *MT1E* gene promotor region. (**D**,**E**) Distinct copies of those two ARE-sequences from the *MT1E* gene were allowed for driving relevant luciferase reporter genes. Each of the ARE-driven reporters—together with expression constructs for Nrf1, Nrf2, or an empty plasmid—were co-transfected into *WT* cells for the indicated times. Then the cells were allowed for recovery from transfection, before the luciferase activity was measured, as shown graphically as fold changes (mean ± SD, *n* = 3 × 3) relative to the background controls (* *p* < 0.05 and ** *p* < 0.01 indicate significant differences with controls). The data were representative of at least three independent experiments, each of which was performed in triplicates. (**F**) A proposed model is provided to give a better explanation of distinctive yet cooperative roles of Nrf1 and Nrf2 in synergistically regulating antioxidant cytoprotective genes against the pro-oxidative stressor *t*BHQ. Of note, the ER-associated Nrf1 was subject to selective topovectorial processing of this protein to yield an active N-terminally-truncated CNC-bZIP factor and then translocate the nucleus before transcriptionally regulating its target genes. Importantly, Nrf1 can act as a brake control for Nrf2’s functionality to be confined within a certain extent, albeit its transcriptional expression is also positively regulated by Nrf2. This is based on the fact that specific loss of *Nrf1α*^−/−^ enables Nrf2 to be accumulated as a hyper-active factor. In addition, *t*BHQ can also trigger a certain yield of ROS in different cell lines, before its stimulation of antioxidant cytoprotective responses mediated by cooperation of Nrf1, Nrf2, and their target genes within a complex hierarchical regulatory network.

Further analysis by flow cytometry unraveled that only a few number of apoptotic cells were indeed examined in *WT* cells that had been intervened with *t*BHQ for 16 h (Figure 6A,B). By contrast, a considerable augment in basal apoptosis of *Nrf1α*^−/−^ cells reached to a much higher rate than that of the other cell lines, but its *t*BHQ-stimulated apoptosis was significantly decreased after intervention of *Nrf1α*^−/−^ cells by this chemical for 4 h to 16 h (Figure 6A,B), to a similar level to that of *t*BHQ-treated *WT* cells. This phenomenon appeared to be almost consistent with the results of changing ROS levels as detected above (Figure 5A,B). These indicate that *t*BHQ can induce antioxidant cytoprotective response against endogenous oxidative stress arising from *Nrf1α*^−/−^ cells, in which putative hyper-expressed Nrf2 may be allowed for a certain extent to ameliorate potential oxidative damage and apoptosis caused by loss of Nrf1α, but could not fully compensate the constitutive loss of Nrf1′s function. Contrarily, no significant differences in basal apoptosis of either *Nrf2^−/−ΔTA^* or *caNrf2^ΔN^* cell lines were observed when compared with that of *WT* cells (Figure 6A,B), but both mutants led to a modest or less increase in *t*BHQ-triggered apoptosis after intervention of *Nrf2^−/−ΔTA^* or *caNrf2^ΔN^* cells, respectively. This indicates that the sensitivity to *t*BHQ cytotoxicity may be weakened by *Nrf2-*deficient mutants, allowing for the resistance of these two cell lines to a considerable extent of pro-oxidative stress. Overall, it could be concluded that both Nrf1 and Nrf2 play distinctive roles in mediating differential antioxidant cytoprotective responses against oxidative stress-induced apoptosis.

This concluding notion is supported by further luciferase reporter assays (Figure 6C), in which the reporter gene was driven by two different ARE-battery sequences existing in the promoter region of human *MT1E* (i.e., *MT1E-ARE1* and *MT1E-ARE2*). The results revealed that the transactivation activity of *MT1E-2×ARE1-luc* was mediated by Nrf1 rather than Nrf2, but no changes in transcriptional expression of *MT1E-2×ARE2-luc* were examined (Figure 6D). However, a significant amplified activity of *MT1E-6×ARE2-luc* was mediated by Nrf2 rather than Nrf1, even although the *MT1E-6×ARE1-luc* was still modestly induced by Nrf1, but not Nrf2 (Figure 6E). Such differences in the gene activity of between *MT1E-2×ARE2-luc* and *MT1E-6×ARE2-luc* may be relevant to their contexts in the reporter gene constructs, but the detailed mechanism remains elusive.

## 4. Discussion

Since oxidative stress was initially formulated by Helmut Sies in 1985 and later redefined by Dean P. Jones in 2006 [46,47,48], an overwhelming number of publications by this conceptual term had been collected within at least 331,795 entries of the PubMed (https://pubmed.ncbi.nlm.nih.gov, accessed on 5 June 2021). Such a perennially vital topic as oxidative stress (and redox signaling) is open to arouse great concerns from researchers in distinct fields, but also is one of the most persistently-existing intractable problems to be addressed for health and disease, particularly in changing environmental conditions. Amongst its merits elicited by evoking biological stress responses, a steady-state redox balance is maintained within certain threshold ranges by cell respiration, aerobic metabolism, and redox switches governing oxidative stress responses [46,47]. However, the pitfalls of oxidative stress can also lead to indiscriminate use of this term as a global concept, but without a clear relation to redox chemistry, in each of the particular cases. For the underlying molecular details, the major role in antioxidant defense is fulfilled by antioxidant enzymes, but not by small-molecule antioxidant compounds (e.g., *t*BHQ), in the cellular biochemical processes.

In all life forms, distinct types of cells can constantly generate a certain amount of ROS (and free radicals) during aerobic metabolism, such that its hormetic effects could be triggered in order to establish normal physiological cytoprotective mechanisms against oxidative damages. Of note, oxidative stress occurs in cells when ROS production overwhelms the natural antioxidant defenses and/or redox controls are disrupted [46,47]. If oxidative stress is over-stimulated for a long period, the resulting damages lead to various pathophysiological conditions which can result in many human chronic diseases—including cancer, diabetes, atherosclerosis, and neurodegenerative diseases [28,49]. Thereby, to combat the excessive production of ROS, all the cells have been evolutionarily armed with a series of innate powerful antioxidant defense systems. Amongst them is a set of essential antioxidant, detoxification, and cytoprotective mechanisms governed by the CNC-bZIP family of transcription factors [12,50,51]. In mammalian cells, Nrf1 and Nrf2 are two principal CNC-bZIP factors to regulate target genes by specific ARE-binding sequences in the promoter regions. To date, a large number of studies on Nrf2 had revealed it functions as a master regulator of antioxidant response and relevant redox signaling [25]. Rather, such versatile Nrf2 acts de facto as a promiscuous, but not essential, player for the optimal ARE-binding to most of its target genes [52], supporting the concluding notion that Nrf2 is dispensable for normal growth and development [26], with no any pathological phenotypes being manifested in its global knockout mice. As a matter of fact, Nrf1, rather than Nrf2, is a living fossil with its ancestral properties, because it shares a highly evolutionary conservativity with SKN-1, Cnc, and Nach factors [51]. Like its ancient homologues [53,54], Nrf1 is topologically dislocated across ER membranes and then processed to give rise to an N-terminally-truncated active factor, similar to Nrf2, before regulating its cognate target genes [55,56,57]. Thus, it is inferable that a unique conserved, indispensable role is fulfilled by Nrf1, but not by Nrf2, in maintaining the steady-state threshold of robust redox homeostasis during healthy life process.

The evidence has been provided in the present study, unraveling differential yet integral contributions of Nrf1 and Nrf2 to synergistic regulation of antioxidant cytoprotective genes at basal and *t*BHQ-inducible expression levels in wild-type (*WT*) cells. Specific knockout of *Nrf1α*^−/−^ leads to severe endogenous oxidative stress as elicited by increased basal ROS levels; this is accompanied by increased ratios of GSSG to GSH and apoptosis. In *Nrf1α*^−/−^ cells, Nrf2 was highly accumulated, but also cannot fully compensate loss of Nrf1α’s function in its basal cytoprotective response against endogenous oxidative stress, even though it had exerted partially inducible antioxidant response as the hormetic effect of *t*BHQ against apoptotic damages. By striking contrast, *Nrf2^−/−ΔTA^* cells were also manifested by obvious oxidative stress, partially resulting from a substantial reduction of Nrf1 in basal and *t*BHQ-stimulated expression levels. However, the inactive *Nrf2^−/−ΔTA^* cells can be still triggered to mediate a potent antioxidant response to *t*BHQ, as deciphered by a significantly decreased ration of GSSG to GSH. Conversely, a remarkable increase of the Nrf1 expression was obtained from the constitutive active *caNrf2^ΔN^* cells, in which neither oxidative stress nor apoptotic damages had occurred, no matter if it was intervened with *t*BHQ. Thereby, distinct yet joint functions of Nrf1 and Nrf2 may be executed through their inter-regulatory effects on cognate genes against oxidative stress (Figure 6F).

Differences in ROS-scavenging activities of SOD and CAT were determined in distinct genotypic cell lines. Basal activities of SOD and CAT were significantly increased by *Nrf1α*^−/−^, *Nrf2^−/−ΔTA^*, or *caNrf2^ΔN^*, when compared to those of *WT* cells. *t*BHQ-inducible SOD activity were marginally elevated in *Nrf1α*^−/−^, *Nrf2^−/−ΔTA^*, but not *caNrf2^ΔN^* or *WT*, cell lines, as accompanied by an exceptional decrease of its activity by 16 h of this stimulation. The modest changes suggest that SOD activity may be monitored by other factors beyond Nrf1 and Nrf2. This notion is also supported by the previous RT-qPCR data [45]. Further evidence also revealed that *t*BHQ-stimulated CAT activity was markedly augmented in *Nrf1α*^−/−^ cells (with hyper-active Nrf2 accumulation), and thereby completely abolished in *Nrf2^−/−ΔTA^* cells. However, basal increased CAT activity was substantially reduced by *t*BHQ intervention of *caNrf2^ΔN^* cells (also with enhanced expression of Nrf1), although this constitutive activator *per se* was unaffected by this chemical. These imply that CAT activity is regulated positively by Nrf2, and also monitored negatively by Nrf1, particularly during *t*BHQ-stimulated conditions. As such, it cannot also be ruled out that loss of the N-terminal Keap1-binding Neh2 domain from Nrf2 to yield *caNrf2^ΔN^* may cause a negative effect on the *t*BHQ-stimulated CAT activity.

As a widely used Nrf2-activator, *t*BHQ can also trigger a certain activating effect on the expression of Nrf1 in *WT* cells, as well in *caNrf2^ΔN^* cells, but this effect is almost totally abolished by *Nrf1α*^−/−^ or *Nrf2^−/−ΔTA^*. Conversely, induction of *Nrf2* expression by *t*BHQ only occurred in *WT* cells alone, but not in *Nrf1α*^−/−^, *Nrf2^−/−ΔTA^*, or *caNrf2^ΔN^* cell lines, although its basal expression levels are significantly augmented in either *Nrf1α*^−/−^ or *caNrf2^ΔN^* cell lines. Together, with our previous data [31,40], these indicate that Nrf1 has an ability to confine Nrf2 within a certain extent, albeit its transcriptional expression is positively regulated by Nrf2, which functions as a limited chameleon activator. Such inter-regulatory effects of both Nrf1 and Nrf2 on antioxidant, detoxification and cytoprotective genes, such as *HO*-*1*, *NQO1*, *GCLC*, *GCLM*, *GSR*, *GPX1*, *TALDO*, *MT1E* and *MT2*, were further determined in distinct genotypic cell lines. As anticipated, the comprehensive experimental evidence has been provided herein, unraveling that *HO*-*1*, *NQO1*, *GCLC*, *GCLM*, *GSR*, and *TALDO* were induced by *t*BHQ stimulation of *WT* cells, but *GPX1*, *MT1E*, and *MT2* were not stimulated or even slightly suppressed by this chemical. By contrast, *Nrf1α*^−/−^ cells were still allowed for *t*BHQ-increased expression of *HO*-*1*, *NQO1*, *GCLM*, and *TALDO*, but with an exceptional decrease of *GPX1*, even though hyper-expressed Nrf2 was roughly unaffected by *t*BHQ. This implies an additional involvement of other transcriptional factors beyond Nrf1 and Nrf2 in mediating these gene response to *t*BHQ as a pro-oxidative stressor. More intriguingly, both basal and *t*BHQ-stimulated expression levels of *MT1E* were strikingly augmented in *Nrf2^−/−ΔTA^*, but not *Nrf1α*^−/−^ or *caNrf2^ΔN^*, cell lines, whereas *MT2* was marginally induced by *t*BHQ in *caNrf2^ΔN^* cells. This finding implies that *MT1E*, but not *MT2*, may serve as an Nrf1-specific target gene, as further evidenced by its relevant reporter assays. Moreover, these was also accompanied by a modest inducible enhancement of GLCM and Nrf1, whereas all other examined genes were, to lesser or no extents, stimulated by *t*BHQ in either *Nrf2^−/−ΔTA^* or *caNrf2^ΔN^* cell lines. These indicated that most of all other examined genes except *MT1E* are regulated primarily by Nrf2, but induction of its transactivation activity by *t*BHQ is also limited by its constitutive loss of the Keap1-binding Neh2 domain in the mutant *caNrf2^ΔN^* factor.

## 5. Concluding Remarks

Dramatic research advances of the past 25 years, since a fascinating discovery by Itoh et al. [23], have witnessed an overwhelmingly preferential option for a sole Nrf2 focus in all relevant fields, whereas Nrf1 was almost totally ignored by nearly all others except for a handful of groups. Such disproportionately biased consequence has resulted in a general misunderstanding of Nrf2 as an only master hub of predominantly regulating antioxidant, detoxification, and cytoprotective genes; regardless of the exciting fact that Nrf1, rather than Nrf2, is highly conserved with those more ancient SKN-1, Cnc, and Nach factors [51], and that it can also fulfill unique indispensable roles for cell homeostasis and organ integrity during the life process. In the present study, together with our previous publications [31,40], Nrf1 and Nrf2 are experimentally evidenced to elicit differential yet integral roles in mediating antioxidant cytoprotective responsive genes against pro-oxidative stress induced by *t*BHQ. The inter-regulatory effects of Nrf1 and Nrf2 on differential expression levels of antioxidant cytoprotective genes—e.g., *HO*-*1*, *NQO1*, *GCLC*, *GCLM*, *GSR*, *GPX1*, *TALDO*, *MT1E*, and *MT2*—as well on the ROS-scavenging activities of SOD and CAT, were determined in depth. The collective results demonstrate that both Nrf1 and Nrf2 can make distinctive yet cooperative contributions to finely tuning basal and/or *t*BHQ-stimulated expression of target genes within their inter-regulatory networks. Overall, Nrf1 can be allowed to act as a brake control for confining Nrf2′s functionality within a certain extent, albeit its transcriptional expression is positively regulated by Nrf2.

## Figures and Tables

**Table 1:** Primer pairs used for the RT-qPCR analysis.

ID	Name	Forward Primers (5′–3′)	Reverse Primers (5′–3′)
60	β-*actin*	CATGTACGTTGCTATCCAGGC	CTCCTTAATGTCACGCACGAT
4776	*Nrf1*	GAAGCCCACCAAGACCGAA	GCCTCTTCCTGTACACTGACC
4780	*Nrf2*	ATATTCCCGGTCACATCGAGA	ATGTCCTGTTGCATACCGTCT
2729	*GCLC*	TCAATGGGAAGGAAGGTGTGTT	TCAATGGGAAGGAAGGTGTGTT
2730	*GCLM*	TCAATGGGAAGGAAGGTGTGTT	CGCTTGAATGTCAGGAATGCTT
2876	*Gpx1*	CAGTCGGTGTATGCCTTCTCG	GAGGGACGCCACATTCTCG
2936	*GSR*	CACGAGTGATCCCAAGCCC	CAATGTAACCTGCACCAACAATG
6888	*TALDO*	GGGCCGAGTATCCACAGAAG	GGCGAAGGAGAAGAGTAACG
1728	*NQO1*	AAGAAGAAAGGATGGGAGGTGG	GAACAGACTCGGCAGGATACTG
3162	*HO-1*	CAGAGCCTGGAAGACACCCTAA	AAACCACCCCAACCCTGCTAT
4493	*MT1E*	ATGGACCCCAACTGCTCTTGCGCCA	ACAGCAGCTGCACTTCTCCGATG
4502	*MT2*	GTGGGCTGTGCCAAGTGT	CAAACGGTCACGGTCAGG

## Data Availability

All data needed to evaluate the conclusions in the paper are present in this publication along with the supplementary information documents that can be found online. The additional data related to this paper may also be requested from the corresponding author on reasonable request.

## Supplementary Materials

The following are available online at https://www.mdpi.com/article/10.3390/antiox10101610/s1, Figure S1: Time-dependent changes in the mRNA expression of distinctive responsive genes to tBHQ; and Figure S2: Different time-dependent effects of tBHQ on ROS level in distinct cell lines.
