# Supplementary material for: Differential Yet Integral Contributions of Nrf1 and Nrf2 in the Human HepG2 Cells on Antioxidant Cytoprotective Response against Tert-Butylhydroquinone as a Pro-Oxidative Stressor
Source: Antioxidants (Basel). 2021 Oct 13;10(10):1610. doi: 10.3390/antiox10101610 (PMC8533631; doi:10.3390/antiox10101610)
Supplement: Supplementary file 1 [file antioxidants-10-01610-s001.zip › antioxidants-1380645-supplementary.pdf]

antioxidants

MDPI

Article

Differential Yet Integral Contributions of Nrf1 and Nrf2 in the Human HepG2 Cells on Antioxidant Cytoprotective Response against *Tert*-Butylhydroquinone as a Pro-Oxidative Stressor

Reziyamu Wufuer, Zhuo Fan, Keli Liu, and Yiguo Zhang *

Laboratory of Cell Biochemistry and Topogenetic Regulation, College of Bioengineering and Faculty of Medical Sciences, Chongqing University, No. 174 Shazheng Street, Shapingba District, Chongqing 400044, China; 20191901703@cqu.edu.cn (R.W.); 18623059592@163.com (Z.F.); 201919021016@cqu.edu.cn (K.L.)

* Correspondence: yiguozhang@cqu.edu.cn

Supplementary Materials

Figure S1

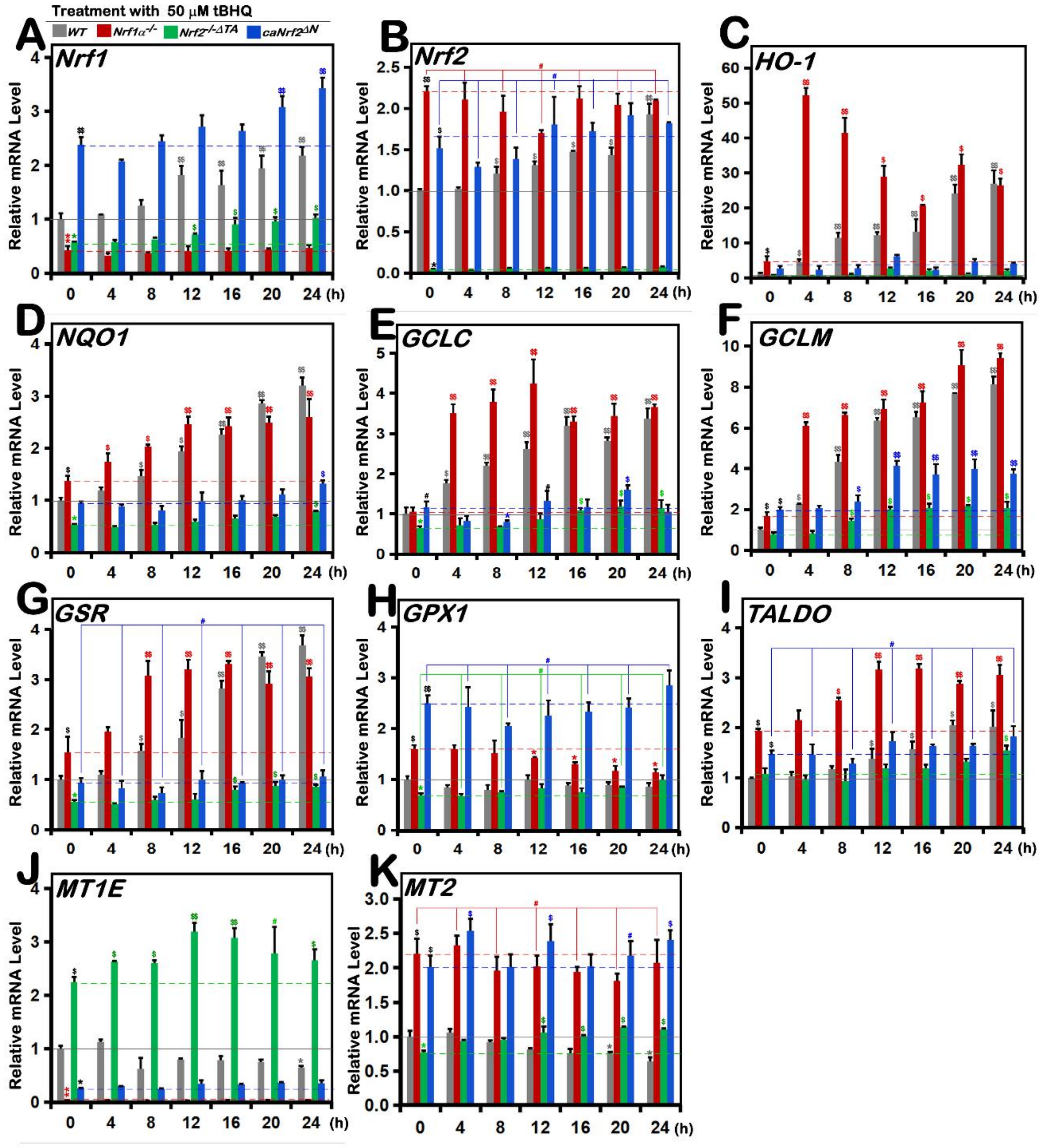

Figure S1: Time-dependent changes in the mRNA expression of distinctive responsive genes to *t*BHQ. For the detailed description, please see the legend of Figure 2 in the main text.

Figure S2

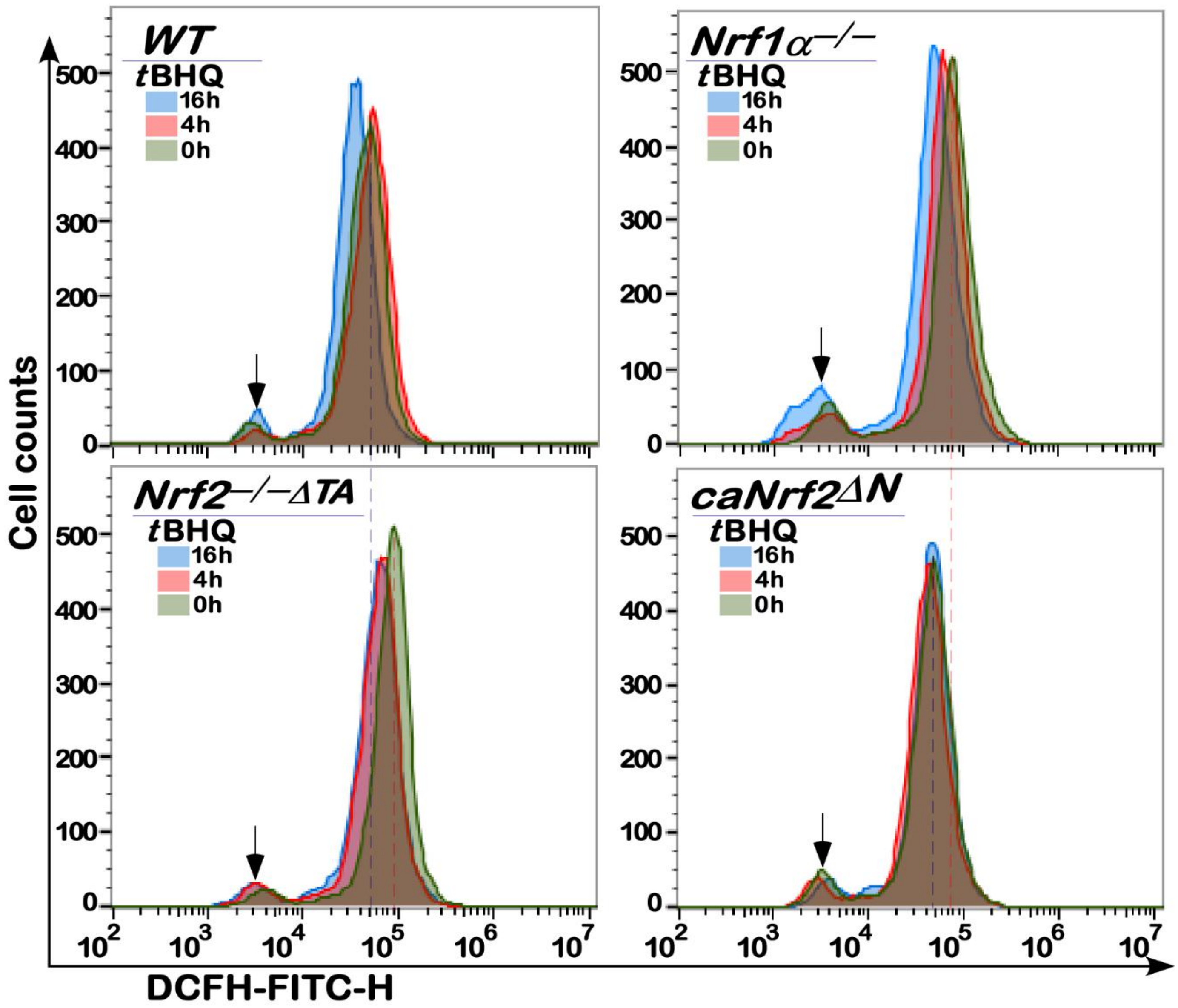

Figure S2. Different time-dependent effects of *t*BHQ on ROS level in distinct cell lines. For the detailed description, please see the legend of Figure 5A in the main text.
